# Intramedullary Nailing Versus Plate Osteosynthesis for Humeral Shaft Metastatic Lesions

**DOI:** 10.7759/cureus.13788

**Published:** 2021-03-09

**Authors:** Marc-Antoine M Ricard, Nikolaos A Stavropoulos, Anas Nooh, Nathalie Ste-Marie, Krista Goulding, Robert Turcotte

**Affiliations:** 1 Department of Orthopaedic Surgery, The Ottawa Hospital, Ottawa University, Ottawa, CAN; 2 Department of Orthopaedic Surgery, General Hospital of Karpenisi, Karpenisi, GRC; 3 Department of Orthopaedic Surgery, McGill University Health Centre, Montréal, CAN

**Keywords:** keywords: intramedullary nail, humeral bone metastasis, plate osteosynthesis, pathologic fracture, humerus fracture

## Abstract

In the event of surgical management of metastases to the humeral shaft, intramedullary nailing (IMN) is often preferred to plate osteosynthesis (PO) fixation despite a lack of consensus. In this study, we hypothesized that plate osteosynthesis will be associated with better functional and pain outcomes, thus better quality of life. Eighteen patients with the diagnosis of humeral shaft metastatic fracture or impending fracture were extracted from a prospective database of 140 metastatic patients collected across three hospitals over a five-year period. Musculoskeletal Tumor Society (MSTS) score, Toronto Extremity Salvage Score (TESS), Quality of Life (QOL) and Brief Pain Inventory (BPI) score were gathered during the year following the surgery. Statistical analysis was performed to compare the mean score differences between the two surgical options at baseline and five follow-up visits. Both treatment options were associated with an increase in functional outcomes based on both MSTS and TESS, and a decrease in pain level. However, no significant difference was found in quality of life and between the two treatment modalities. Thus, based on our results, a similar improvement in functional status and pain level can be achieved surgically by either intramedullary nailing or plating osteosynthesis.

## Introduction

Studies show that up to 20% of all bony metastases are located in the upper extremities and more specifically, 50% of those are found in the humerus [1]. Unfortunately, these bone lesions are sometimes associated with functional impairments and risk of fracture. Options available for the management of those lesions are the following: nonsurgical approach with utilization of chemotherapy, radiation therapy and splinting, internal fixation of the bone with plate and screws or nail with or without debulking of the tumor, resection of the diseased bony segment and reconstruction of the structural defect with segmental endoprosthetic implant or exceptionally, allograft [2,3]. The optimal treatment option is determined by the extent and location of the metastases while accounting for the estimated life expectancy and the anticipation of local tumor response to adjuvant therapies. When facing short life expectancy or multiple co-morbidities, non-surgical options, such as radiotherapy alone, are usually preferred over surgery. Surgical management is thought to be most efficient in restoring function and reducing pain, especially when dealing with a fracture. Metastatic bone lesions have limited potential for healing, thus quick relief of pain and immediate restoration of function is imperative in this population of limited life expectancy. Guidelines are available to the case selection that would most likely benefit from surgery such as the Mirel’s score for predicting the risk of pathologic fractures [4]. In the case of humeral metastatic impending or established fracture and when repairing of the bone is chosen, intramedullary (IM) nailing might be the preferred option based on its mechanical advantages and for its whole bone protection [3,5,6]. Studies reported that IM nailing (IMN) was preferred in more than 90% of the cases in comparison to the other treatment modalities [5,7]. As an alternative, plate osteosynthesis is an option that leads to a much more extensive surgical exposure, higher blood loss and weaker construct which may affect the overall outcome in case of complications such as failure of the bone to unite or local progression of tumor despite treatment [8]. 

Some studies have shown that either technique can restore functional status to a similar extent, while others concluded that nailing was a better choice compared to plate osteosynthesis with optimal functional results and lesser complications associated with it [7-10]. In the trauma literature, antegrade nailing of the humerus has been reported with slower recovery of shoulder function and greater pain compared to plating [11]. It remains unclear which surgical procedures should be favored, based on previous studies, to ensure biomechanical stability and function restoration even without fracture healing and to provide quick improvement of pain.

The aim of this study was to determine which of these two procedures allowed maximal improvement of function and quality of life while decreasing the level of pain in patients with humeral shaft fractures or impending fracture from metastatic lesions. We hypothesized that plate osteosynthesis, compared to intramedullary nailing, would be found superior due to its lesser impact on the shoulder joint and rotator cuff and that complication rates would be similar.

## Materials and methods

We conducted a multicentered prospective data collection of long bone metastasis managed surgically that led to a 140-patient database between 2014 and 2017. Three centers participated - Hôpital Maisonneuve-Rosemont (HMR), Centre Hospitalier Universitaire de Québec (CHUQ) and McGill University Health Center (MUHC). Among data collected were patient demographics, tumor characteristics, surgical management and complications. The functional outcome (evaluated by both Musculoskeletal Tumor Society [MSTS] and Toronto Extremity Salvage Score [TESS]), pain level (evaluated by Brief Pain Inventory [BPI]) and quality of life (evaluated by the Quality of Life in the Life-Threatening Illness-Patient questionnaire [QOLLTI-P]) were also recorded both pre- and post-surgery [12-16]. Postoperatively, patients were assessed at two, six, 12, 26, and 52 weeks. From this study of surgically managed long bones metastasis, we specifically extracted the patients who presented with humeral diaphyseal lesions. The choice of performing either plate osteosynthesis or intramedullary nailing was made by the surgeon based on the clinical presentation of the case and the preferences of both the patient and the surgeon. In total, 18 patients met the study criteria. We first assessed if the two groups differed at baseline by conducting a two-sample t-test for each evaluation. Then the repeated measurements of functional outcomes, quality of life and pain level across the surgical groups were analyzed using a two-way repeated measures analysis of variance (ANOVA). The repeated measures factor was follow-up visits and the between-groups factor was surgical groups. Statistical analysis was performed using Stata 14 (StataCorp, College Station, TX, USA; 2015).

## Results

Patients of both groups, IM nail and plate osteosynthesis, were found not statistically different regarding gender, age, comorbidities and adjunct therapies except for cement filing where two out of eight patients (25%) with IM nail procedure received cement compared to nine out of 10 patients (90%) with plating (Table 1).

**Table 1:** Demographics and clinical characteristics of patients with humeral shaft metastases Plate=plate osteosynthesis, DM2= Diabetes 2, HTN=hypertension, DM1= Diabetes 1, MI= myocardial infarction, NeoRT=neoadjuvant radiotherapy, Cement=cement bone filing, AdjCTx= adjuvant chemotherapy, NeoCTx= neoadjuvant chemotherapy, AdjRT= adjuvant radiotherapy

Patient	Gender	Age	Surgery	Comorbidities	Site of primary tumor	Complications	Other treatment modalities
1	M	57	IM nail	Smoking	Lung	Completion impending fracture intra-operative	NeoRT
2	M	77	IM nail	DM2, HTN	Unknown	Bedsores	No
3	F	69	IM nail	No	Lung	Local progression	No
4	M	63	IM nail	no	Myeloma	No	No
5	M	84	IM nail	HTN	Prostate	No	NeoRT
6	F	76	IM nail	HTN	Lung	No	No
7	M	75	IM nail	DM1, HTN	Kidney	No	Cement
8	F	61	IM nail	No	Breast	No	Cement
9	F	73	Plate	No	Lung	No	No
10	M	67	Plate	No	Kidney	No	Cement
11	F	41	Plate	No	Unknown	No	Cement, AdjCTx
12	F	63	Plate	HTN, MI	Myeloma	No	Cement, NeoRT, NeoCTx
13	M	59	Plate	DM2, HTN	Myeloma	Radial nerve injury + Recurrent Drainage hematoma	Cement
14	F	70	Plate	No	Myeloma	No	Cement
15	M	56	Plate	No	Myeloma	No	Cement
16	M	82	Plate	No	Lung	No	Cement, AdjRT
17	F	59	Plate	No	Lung	Local recurrence	Cement
18	M	42	Plate	Smoking	Myeloma	No	Cement

Complications recorded in both types of fixation included: one systemic and one local complication (bedsores post-operatively and fracture upon nail insertion) for the IM nailing group and two local complications (one intra-operative fracture and one hematoma that led to a radial nerve palsy postoperatively) following plate osteosynthesis. One patient with plate osteosynthesis and cement experienced local recurrence at 26 weeks and one patient with nail demonstrated local progression at 12 weeks. In the latter radiotherapy wasn’t performed despite recommendations. Follow-up with plate osteosynthesis included a patient who was only seen at two weeks and one with last visit at six weeks. Four others completed visit at 26 weeks while the two other patients completed the one year study. For IM nail, one patient had only follow-up at two weeks, one patient had his last follow-up at six weeks, two at 12 weeks, one at 26 weeks and five patients completed the 52-week follow-up. Additionally, only half the patients (four) with IM nail were alive at 52 weeks compared to seven (70%) with plate.

**Figure 1:**
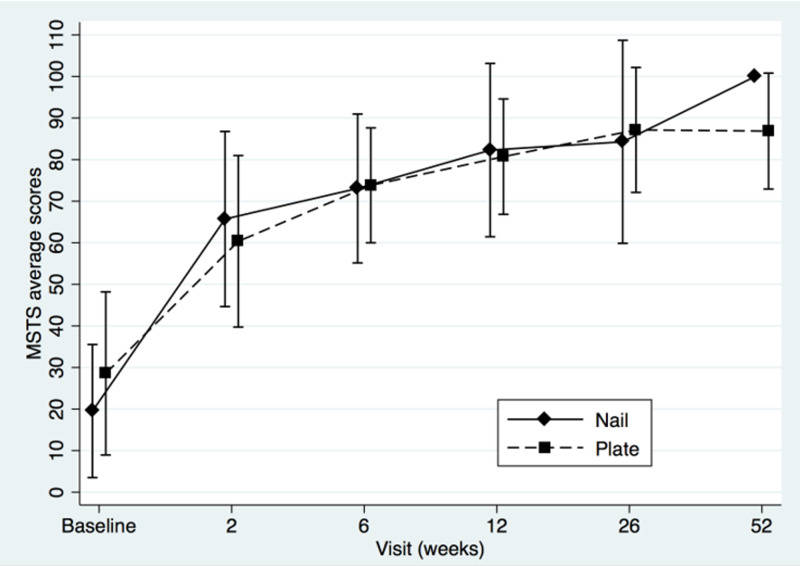
Musculoskeletal Tumor Society (MSTS) mean scores of intramedullary nail and plate osteosynthesis procedures at baseline and post-surgery

**Figure 2:**
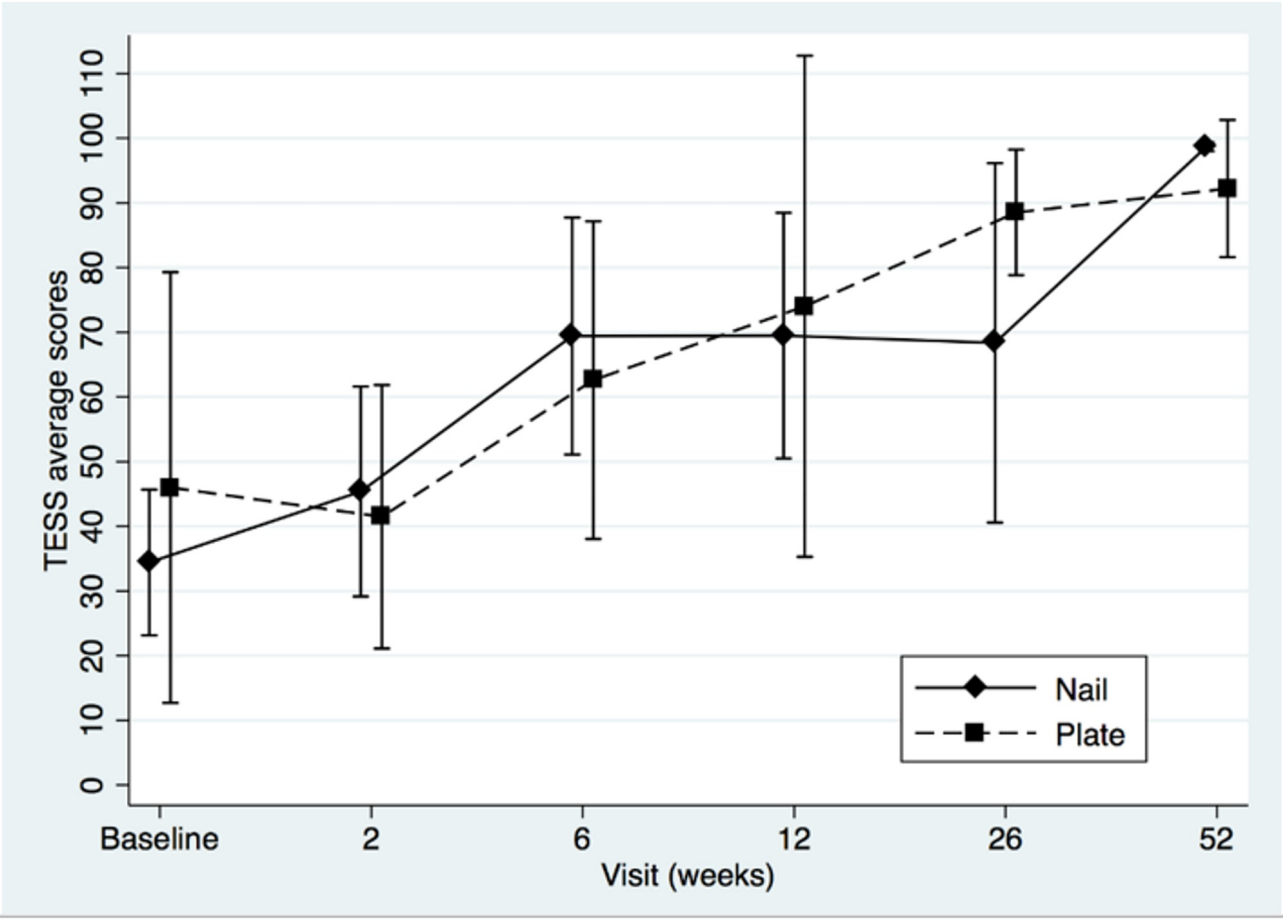
Toronto Extremity Salvage Score (TESS) mean scores of intramedullary nail and plate osteosynthesis procedures at pre- and post-surgery

**Figure 3:**
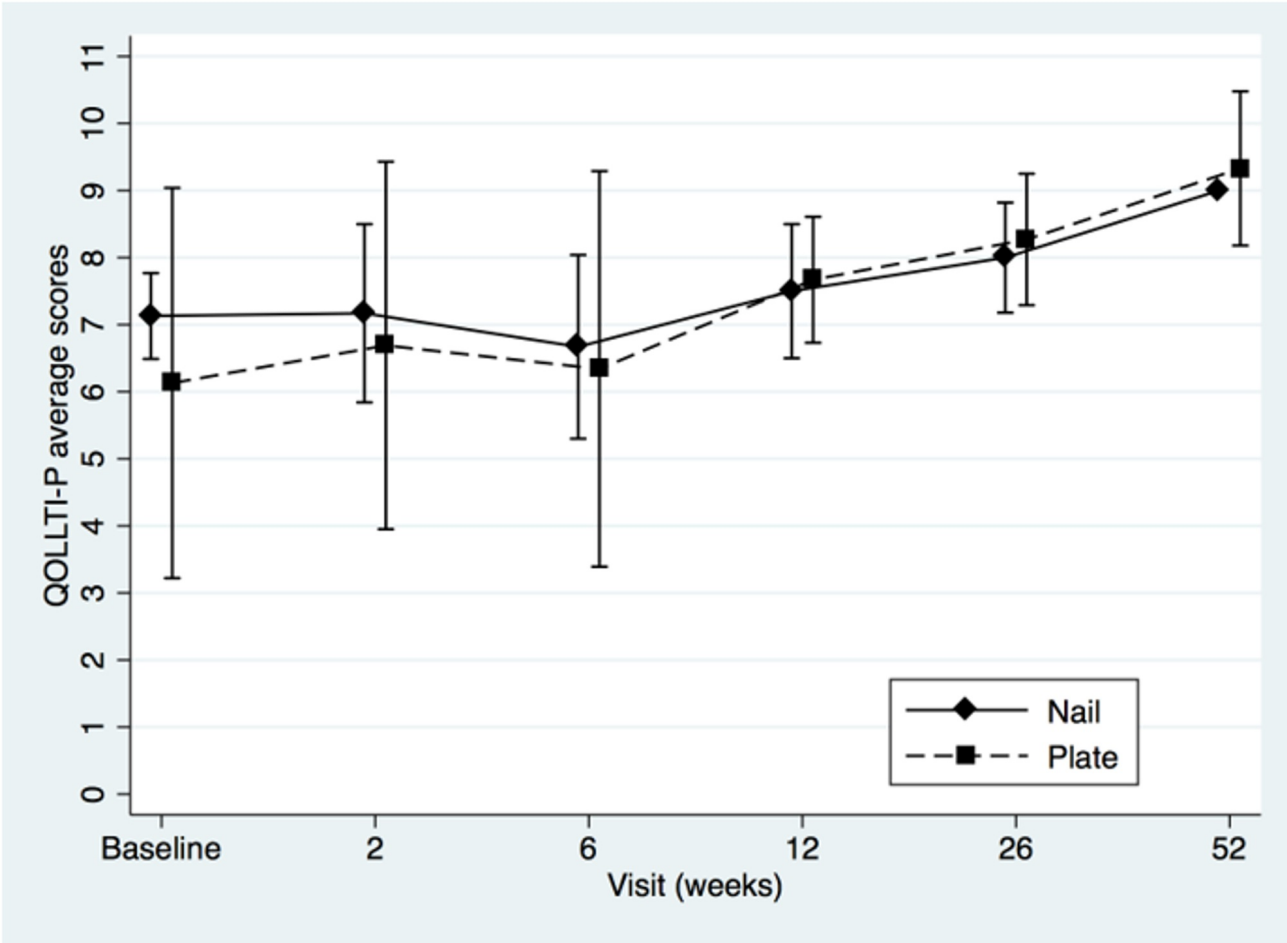
Quality of Life in Life-Threatening Illness-Patient (QOLLTI-P) questionnaire mean scores of intramedullary nail and plate osteosynthesis procedures at pre- and post-surgery

**Figure 4:**
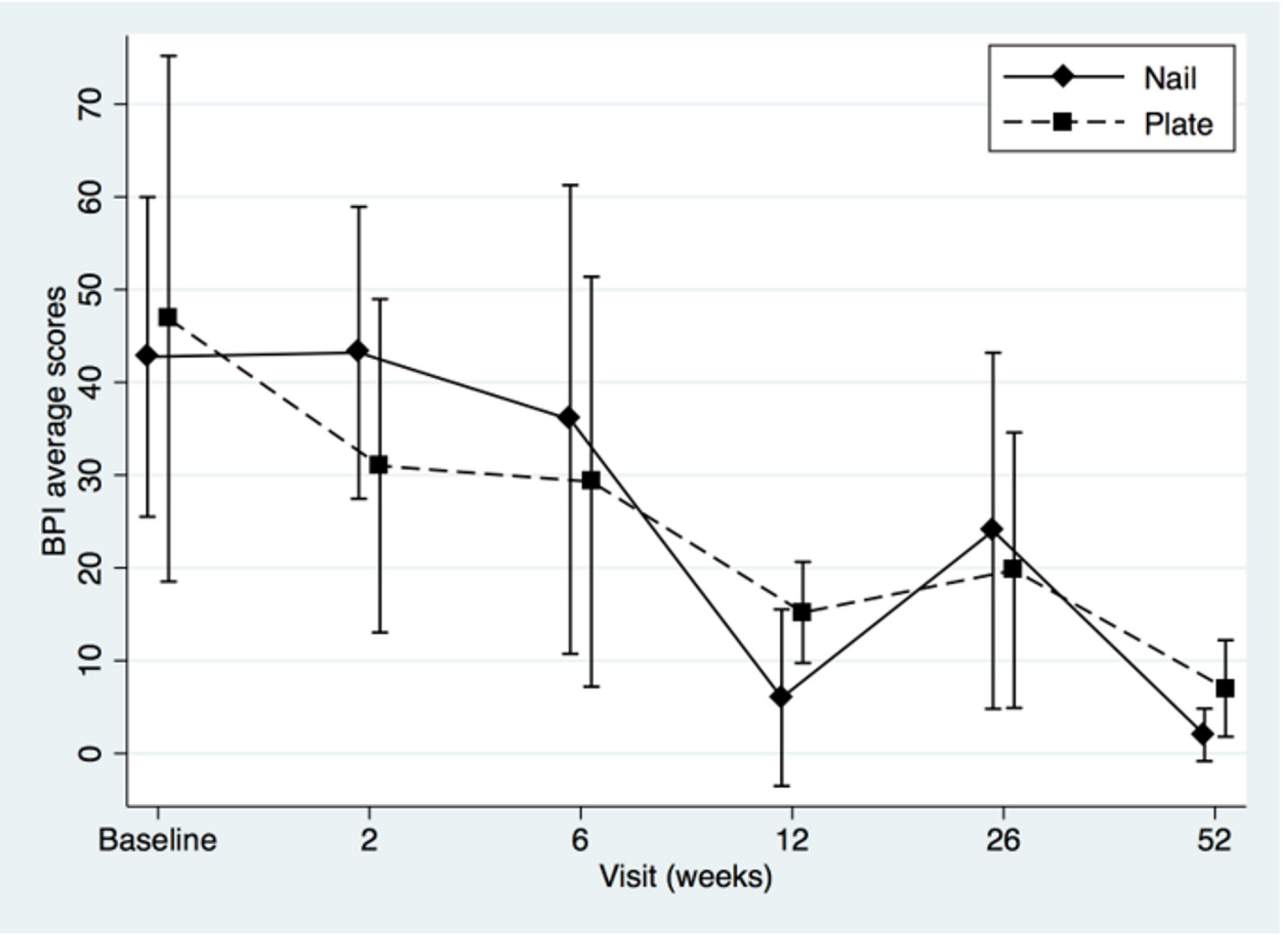
Brief Pain Inventory (BPI) mean scores of intramedullary nail and plate osteosynthesis procedures at pre- and post-surgery

Two-sample t-tests showed that the groups did not differ at baseline (MSTS, t=-0.92, p=0.38; for TESS t=-0.87, p=0.40; for QOLLTI-P, t=0.94, p=0.36; and for BPI t=-0.34, p=0.74). Patients’ MSTS, TESS, quality of life and pain level mean scores were plotted to compare IM nail with plate osteosynthesis fixation (Figures 1-4).

Both treatments showed definite progressive increase in functional outcomes and quality of life scores over time. The BPI score (Figure 4) seemed to favor plating for pain at two weeks, but this difference was not statistically significant (t=1.29, p=0.22). The modest sample size (n=13) decreased statistical power and a post hoc power analysis revealed that, using the means and standard deviations known of this group at this specific follow-up, a sample of approximately 64 (32 in each group) would be needed to obtain statistical power at the recommended .80 level and alpha of 0.05. We performed a repeated-measures ANOVA to determine the difference in functional outcomes, quality of life and pain level between the two groups over time, using a conservative F-test for the interaction between time and treatment group. The Box’s conservative correction for F-test was used to adjust the degrees of freedom for deviations from sphericity, however statistical analysis showed no significant difference at any time between the two treatment options: for MSTS, F(5,38)=0,40, p=0,55; for TESS F(5,33)=1,02, p=0,35; for QOLLTI-P, F(5,37)=0.72, p=0.42; and for BPI F(5,36)=0.45, p=0.52.

## Discussion

**Figure 5:**
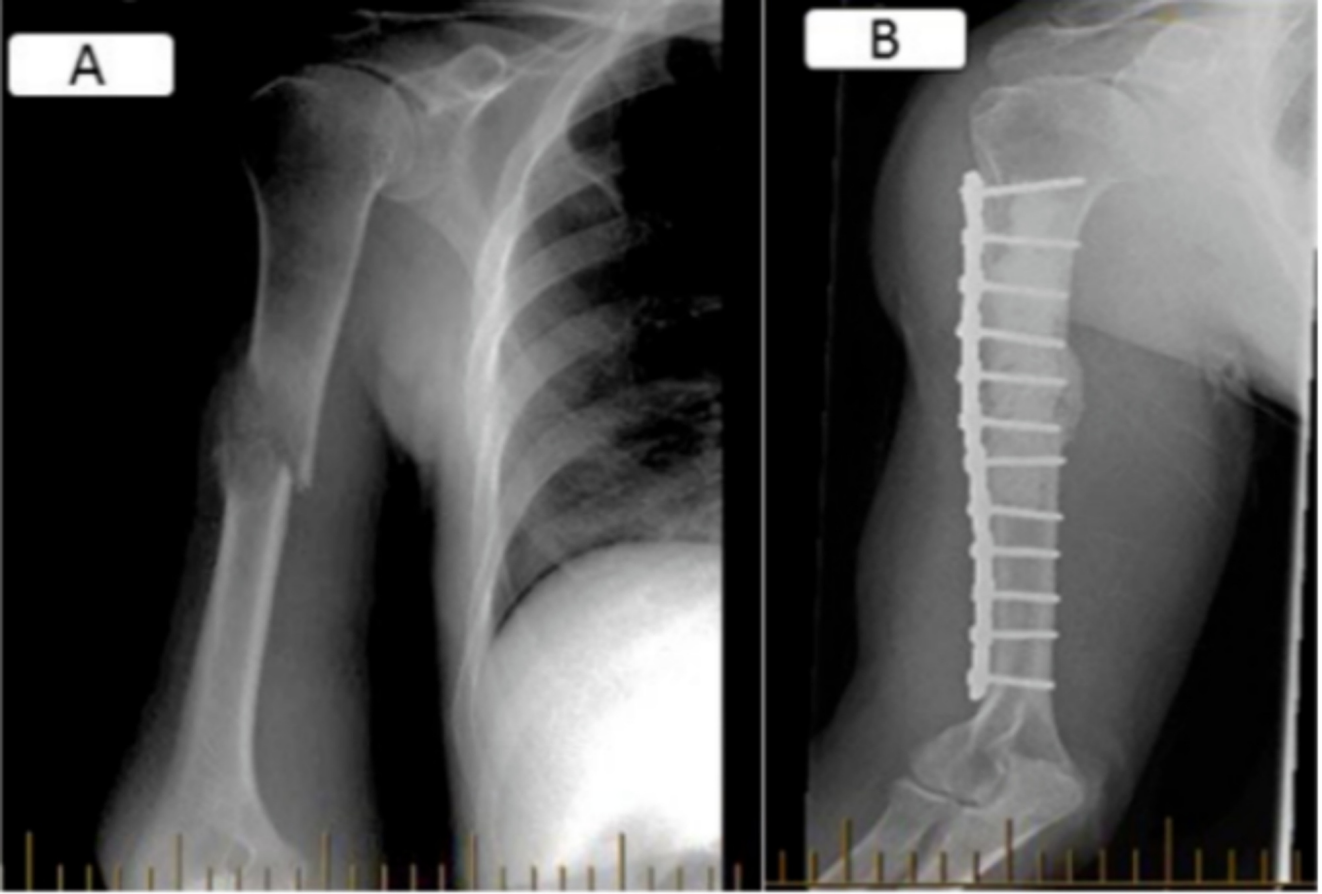
AP views of a right mid diaphyseal humerus metastatic lesion (A) managed with plate osteosynthesis and cementoplasty (B).

**Figure 6:**
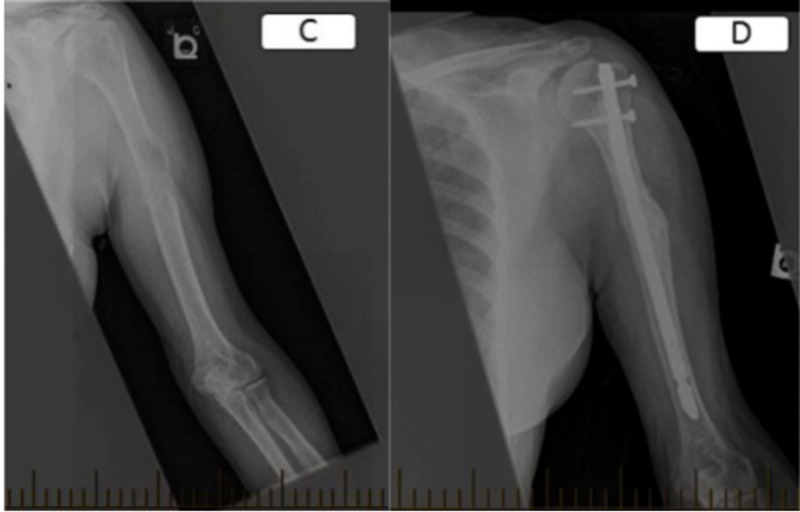
AP views of a left mid diaphyseal humerus metastatic lesion from adenocarcinoma (C) managed with open biopsy and IM nailing (D).

Intramedullary nailing for metastatic disease involving the diaphysis of long bones is often preferred for its biomechanical superiority and for its limited soft tissue violation. Nevertheless, some authors favor fixation with plate and screws over IMN for the humerus to minimize the impacts on rotator cuff and shoulder stiffness that result from the nail’s point of insertion (see Figure 5 and Figure 6). The wider surgical exposure required with the plate procedure being thought to be of lesser consequences. This small series suggests no superiority of either types of fixations when evaluating the functional status, pain level and quality of life after surgery. 

Our findings are in line with retrospective studies that also concluded at no difference between nailing and plating for function [9,17]. It does however differ from others which found a greater improvement in function with intramedullary nailing compared to plate osteosynthesis [2,5,7,8,18,19]. Table 2 outlines available studies on this topic. Our experience was no different from other studies that showed similar rates of complications between the two treatment modalities [19]. Nevertheless, we experienced a nerve injury in the plate osteosynthesis group. Nerve injuries are, however, not reported differently between both treatment modalities [11,17]. However, complications are hard to assess because they depend on many factors such as the surgeon’s experience, the patients themselves, the equipment used, etc. The literature is very unspecific on this subject, ranging from same rate of complications to worse complications in either IM nailing or plate osteosynthesis [20].

**Table 2:** Publications and patient’s characteristics on metastatic humeral fractures IMN = intramedullary nail,  PO = plate osteosynthesis,  PR = prosthetic reconstruction,  AP = angle plate, BP = Bundle Pinning, C = Cementoplasty

Author, year	Study Design	Operative treatment modalities	Total number of fractures	Number IMN (humeral shaft)	Number PO (humeral shaft)	Complication rate (%)	Comparison IMN vs PO (%)	Functinonal score (%)
Korkala et al. 1991 [22]	Retrospective	IMN, PO, PR, AP	52	5	4	Local Surgical: - Systemic Surgical: - Fixation Failure: 2 Re-operation: 1	Failure IMN: 0 ; PO: 2	-
Dijkstra et al. 1994 [10]	Retrospective	IMN, PO	38	18	20	Local Surgical: 5 Systemic Surgical: 7 Fixation Failure: 4 Re-operation: -	Local: IMN: 1; PO: 4 Systemic: IMN: 3; PO: 4 Failure : IMN: 3; PO: 1	Normal function: IMN: 9 (50); PO: 11 (55); Total: 20 (52)
Gebhart et al. 2001 [7]	Retrospective	IMN, PO, PR	56	38 (no distinction neck vs shaft vs head)	1	Local Surgical: 0 Systemic Surgical: 0 Fixation Failure: 1 Re-operation: 1	Failure IMN: 1; PO: 0	Normal function Total: 30 (59) IMN: 79%
Talbot et al. 2005 [25]	Prospective	IMN, PO, PR	67	36	5	Local Surgical: 6 Systemic Surgical: 6 Fixation Failure: 1 Re-operation: 3 (4.5)	-	-
Sarahrudi et al. 2009 [20]	Retrospective	IMN, PO	41	20	21	Local Surgical: 6 (15) Systemic Surgical: 0 Fixation Failure: 5 Re-operation: 2	Failure IMN: 3 (15); PO: 2 (10) Local IMN: 0; PO: 6	Better in IMN since less cases of radial nerve injury
Wedin et al. 2012 [8]	Retrospective	IMN, PO, other	214	117	11	Local Surgical: 5 (2) Systemic Surgical: - Fixation Failure: 20 (9) Re-operation: (9)	Failure: IMN: 8 (7); PO: 2 (22) Reoperation: IMN: 5 (7); PO: 11 (22)	-
Thai et al. 2016 [19]	Retrospective	IMN, PO, PR	96	37	2	Local Surgical: 8 Systemic Surgical: 0 Fixation Failure: 1 Re-operation: 1	Local IMN: 5; PO: 3 Re-operation IMN: 1(2); PO: 0	-
Moon et al. 2016 [6]	Retrospective	IMN	40	40	-	Local Surgical: 2 Systemic Surgical: 1 Fixation Failure: - Re-operation: -	-	-
Kim et al. 2016 [23]	Prospective	IMN	70	43 with cement, 27 without	-	Local Surgical: 3 Systemic Surgical: 5 Fixation Failure: 0 Re-operation: 0	-	-
Choi et al. 2016 [24]	Retrospective	IMN	32	32 (with head and neck)	-	Local Surgical: 1 Systemic Surgical: 0 Fixation Failure: 0 Re-operation: 0	-	Mean: MSTS: 27,7 KPS scale: 75,6
Bayram et al. 2019 [18]	Retrospective	IMN	56	56	-	Local Surgical: 2 Systemic Surgical: 3 Fixation Failure: 0 Re-operation: 0	-	ECOG 1 (=9), 2 (=20), 3 (=9), 4 (=14)
Moura et al. 2019 [5]	Retrospective	IMN	86	86	-	Local Surgical: 3 Systemic Surgical: 0 Fixation Failure: 1 Re-operation: 0	Failure IMN 1	MSTS 72.6% post op
De Geyer et al. 2020 [3]	Retrospective	IMN, PO, PR, BP, C	112	77	12	Local Surgical: 10 Systemic Surgical: 12 Fixation Failure: 4 Re-operation: 7	Failure IMN 2; PR 2	4 stage function created by authors

Although we expected the plate osteosynthesis to provide a better overall outcome mainly due to the shoulder’s anatomical modalities sparing that it provides, at least in the early postoperative period, both surgical procedures not only displayed similar results but also a similar pattern of improvement at each step of the follow-up visits. We believe however that cement, used mostly with plates (seven out of eight plates and two out of 10 IM nails) has an important role in minimizing fixation failure. This goes along with many other studies that showed benefits to cementing [18,21-24]. In Table 2, we listed the publications and patient's characteristics on metastatic humeral fractures available in the literature.

The strengths of this study lie in its prospective data collection and early assessments of outcomes to confirm quick improvement in the overall condition of the patients. Both are unique in literature. Metastases were all located in the diaphysis to avoid bias in treatment option. Metaphyseal or epiphyseal metastasis to the humerus, such as humeral head and neck were excluded as they are usually managed with periarticular plates or endoprosthesis instead of nail as the latter provides very limited fixation of the epiphyseal fragment. Except for the MSTS which is not validated even if used widely, all the other tools were (TESS, QOLTI-P, and BPI). Our study revealed another interesting point about the correlation between the quality of life and function over time. As seen in Figures 1, 2, and 3, both values (function and quality of life) increase over time while Figure 4 shows the progressive decrease in pain after the surgery. From our literature review, no previous study reported specifically on patients’ quality of life before and after surgery for surgical diaphyseal humeral metastatic bone.

One of the main points reported by other studies was that intramedullary nailing could provide a more durable and stable fixation allowing thus earlier mobility. According to our study, no such claim can be supported for humerus as the quality of life after both procedures was similar and remained similar throughout the follow-up time.

Our study has several limitations. First, it has a small number of patients and is thus likely underpowered to answer the question without doubt. Among the 140 patients that underwent surgery in the original series, only 18 met the inclusion criteria. This also reflects the somewhat rare incidence of specific metastatic diaphyseal humeral bone disease necessitating surgery. Moreover, some patients decided to undergo conservative treatment for many reasons, thus decreasing the study sample. Cases that were managed non-operatively were not included and neither compared with our two surgical groups. There was also selection bias in management of the lesion relating to surgeons’ preferences. No specific shoulder scoring system was used since this study's cases were extracted from a larger prospective study that was designed to address outcomes from various long bones such as femur and tibia and not specifically the area of our interest. 

Prospective studies involving the metastatic bone population are uncommon and difficult to conduct [25-27]. Early loss to follow-up is frequent due to the progressive nature of the disease and difficulty for advanced cancer patients to attend follow-up visits. The life expectancy of these patients is often found to be shorter than the planned follow-up period, thus emphasizing the need for quick recovery and short-term assessment. Only two of the eight patients with IM nailing provided results at 52 weeks. This also gives perspective to the value of data retrospectively collected which makes the essential of reported series. According to our calculation, the design of a prospective study with strong statistical power appears unrealistic.

## Conclusions

This study brings another point of view on the use of intramedullary nail over plate osteosynthesis for diaphyseal metastatic bone lesion of the humerus. Based on our study, regarding function, quality of life, and pain, both provided similar outcomes. One should thus individualize, until proven otherwise, the type of osteosynthesis to perform based on the characteristics of the patients, the lesion or the fracture and on both patient’s and surgeon’s preferences.
